# Texture Analysis of Cartilage Repair Tissue Maturation: Comparison of Two Cartilage Repair Methods and Correlation with MOCART 2.0

**DOI:** 10.1177/19476035241313047

**Published:** 2025-01-29

**Authors:** Veronika Janacova, Pavol Szomolanyi, Diana Sitarcikova, Alexandra Kirner, Siegfried Trattnig, Vladimir Juras

**Affiliations:** 1 High-Field MR Centre, Department of Biomedical Imaging and Image-Guided Therapy, Medical University of Vienna, Vienna, Austria; 2 CD Laboratory for MR Imaging Biomarkers (BIOMAK), Vienna, Austria; 3 Institute of Measurement Science, Slovak Academy of Sciences, Bratislava, Slovakia; 4 TETEC Tissue Engineering Technologies AG, Reutlingen, Germany; 5 Austrian Cluster for Tissue Regeneration, Ludwig Boltzmann Institute for Experimental and Clinical Traumatology, Vienna, Austria; 6 Institute for Clinical Molecular MRI in the Musculoskeletal System, Karl Landsteiner Society, Vienna, Austria

**Keywords:** Articular Cartilage, Tissue, Magnetic resonance imaging, Diagnostics, Knee, Joint involved, Cartilage repair, Repair, Radiomics

## Abstract

**Objective:**

The objective of this study was to assess the maturation of matrix-associated autologous chondrocyte transplantation (MACT) grafts up to 2 years after the surgery using gray-level co-occurrence matrix (GLCM) texture analysis of quantitative T_2_ maps, compare the results with the microfracturing technique (MFX) control group, and relate these results to the morphological MOCART 2.0 score.

**Design:**

A subcohort of 37 patients from prospective, multi-center study underwent examination on a 3T MR scanner, including a T_2_ mapping sequence at 3, 12, and 24 months after surgery. Changes between the time-points in the mean T_2_ values and 20 GLCM features extracted from T_2_ maps were assessed in repair tissue, tissue adjacent to the repair site, and the reference cartilage for both procedures.

**Results:**

Significant correlations were found between the MOCART 2.0 and GLCM features for both surgical procedures. There were no significant differences between MACT and MFX. We identified significant intra-group changes in T_2_ and autocorrelation (3M-12M: *P* = 0.002; 3M-24M: *P* = 0.004), dissimilarity (3M-24M: *P* = 0.01), homogeneity (3M-24M: *P* = 0.013), and correlation (3M-24M: *P* = 0.036), sum average (3M-12M: *P* = 0.001; 3M-24M: *P* = 0.002), and information measure (3M-24M: *P* < 0.001) in the MACT repair tissue. MACT models revealed differences in GLCM between all combinations of ROI types at almost all time-points. In the case of MFX, the significant differences were mainly between repair and reference tissue at 12 months.

**Conclusion:**

Texture analysis provides a useful extension to T_2_ mapping. Texture features are correlated to the morphological outcome and reveal differences in the process of maturation between MACT and MFX.

## Introduction

The articular cartilage, a highly specialized tissue that facilitates joint motion, has a limited capacity for self-healing because it is an avascular and aneural tissue.^1-3^ Defects resulting from trauma can result in debilitating joint conditions, such as early osteoarthritis (OA).^4^ For young adults, joint replacement is not an option, as prostheses can wear out early after approximately 10 years.^5^

In cases where conservative treatment is not effective and lesions are small, minimally invasive techniques based on the stimulation of bone marrow cells, such as microfracturing (MFX) or cartilage restoration techniques, such as matrix-associated autologous chondrocyte transplantation (MACT), are used.^6-10^ Previous research has indicated that the outcome of cartilage repair surgery can vary from hyaline-like tissue to mixed fibrous-hyaline tissue or fibrous tissue with poorer bio-mechanical qualities, which are typically seen after MFX,^11-13^ but problems or suboptimal results can arise from both MFX and MACT.^14,15^

To avoid invasive procedures during the monitoring of patients after cartilage repair surgery, the Magnetic Resonance Observation of Cartilage Repair Tissue (MOCART)^16^ score and MOCART 2.0^17^ score have been introduced. These scores evaluate the quality of cartilage repair by examining factors such as surface of the repair tissue, subchondral bone, defect filling, repair tissue signal intensity, and integration with the surrounding cartilage. However, the gray-level co-occurrence matrix (GLCM)^18^ of collagen-specific T_2_ maps is currently being explored as a quantitative tool with which to assess cartilage repair tissue maturation.

The GLCM identifies the co-occurrence of signal intensities at a designated offset within an image. The matrix then allows the extraction of various quantitative features. These GLCM features (second-order image statistics) reveal linear and quadratic relationships among pixel pairs. As degradation of cartilage matrix leads to increased water mobility and subsequent T_2_ elevation,^19^ differences in T_2_ distributions are often visible on T_2_ maps. GLCM offers new insights into cartilage structure when combined with T_2_ values of cartilage regions. GLCM has already been explored in recent years in the context of detecting early osteoarthritic (OA) changes,^19-27,28^ or in cartilage maturation assessment.^29^

Because GLCM features are handcrafted, calculation parameters must be based on expertise and the problem at hand. The displacement distance (*d*) determines the distance between neighboring pixels,^18^ and quantization into eight, 16, or 32 gray levels simplifies the calculation. It has been shown that 16 gray levels are usually adequate.^30^ Considering the nonhomogeneous cartilage structure, the angle of GLCM calculation is crucial. This was addressed in an earlier study.^31^ To address nonrectangular ROIs, studies have implemented ROI flattening for GLCM reproducibility.^25,32^

The objective of this study was to assess MACT graft maturation up to 2 years after the surgery using GLCM texture analysis of quantitative T_2_ maps, compare the results to the MFX control group, and relate these results to the morphological MOCART 2.0 score.

## Methods

### Study Design and Patient Baseline Characteristics

Ethical approvals for this study were obtained separately for each individual participating and each participant gave written, informed consent. This article covers a prospective, multi-center, randomized, controlled, open-label (blinded MRI reading), phase III study comparing the efficacy and safety of MACT using NOVOCART 3D plus (TETEC—Tissue Engineering Technologies AG, Reutlingen, Germany) versus MFX in patients with focal cartilage defects of the knee. The clinical trial was registered under EudraCT number 2011-005798-22.

Patients, between 18 and 65 years of age, or minors at least 14 years old with a closed epiphyseal growth plate, were randomly assigned to either the MACT or the MFX group in a 2:1 ratio. Eligible participants included both males and females with localized articular cartilage defects in the femoral condyle or trochlea of the knee, graded as III or IV according to the International Cartilage Repair Society (ICRS) classification. Enrollment criteria specified a maximum of two defects, with the total defect size restricted to a maximum of 6 cm² and a minimum of 2 cm². In case of a pediatric patient (14-17 years old), the confirmation of closure of epiphyseal growth plate of the index knee by x-ray or MRI was required. The 2:1 randomization ratio was specified in the original clinical trial registration.

The overall patient cohort (*n* = 262) comprised 189 males (72.1%) and 73 females (27.9%). The average age of the patients was 39.9 ± 10.6 years. The majority of participants (233 patients, 88.9%) had a solitary lesion, while 29 patients (11.1%) presented with two lesions. The majority of cartilage lesions in both treatment groups originated from trauma (77.4% in the MFX group and 78.3% in the MACT group). Both the total lesion size (MFX group: 3.6 ± 1.3 cm²; MACT group: 3.8 ± 1.4 cm²) and the mean size of the larger lesion (MFX group: 3.5 ± 1.2 cm²; MACT group: 3.6 ± 1.2 cm²) were comparable between the two treatment groups. The MRI substudy comprised 110 patients, (MACT: 75 patients; MFX: 35 patients).

### MRI Examination

Patients underwent MRI examinations at 3 months (3M), 12 months (12M), and 24 months (24M) after surgery. Only 27 MACT and 10 MFX patients had T_2_ maps available for all three time-points. The 3T MRI protocol consisted of morphological imaging protocols (three-dimensional proton density-weighted spin-echo sequence, and two-dimensional proton-density, T_1_- and T_2_-weighted fast-spin echo sequences) and sagittal T_2_ mapping multi-echo spin echo sequences (slice thickness: 3 mm; slice spacing: 3.3 mm; repetition time: 2000 ms; echo times (ms): 12.5, 25, 37.5, 50, 62.5, 75, 87.5, 100; averages: 1; flip angle: 90° and 180°; acquisition matrix: 320 × 256; field of view: 16 cm × 16 cm; total acquisition time: 10 minutes 36 seconds). T_2_ mapping was performed off-line in MATLAB R2019a (MathWorks, Natick, MA) using mono-exponential, two-parametric least-squares decay fitting.

### Evaluation of MRI Images

**Figure 1.**
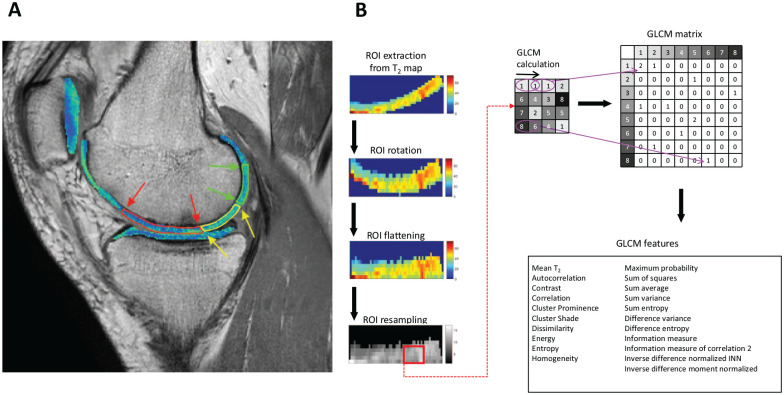
(**A**) Regions of interest (ROIs) selection. Each tissue type is delineated by ROI bounds and arrows. Red: repair tissue, yellow: adjacent tissue, green: reference tissue. (**B**) ROI analysis pipeline.

Morphological images were evaluated by an expert radiologist (S.T.) using the MOCART 2.0^17^ score for semi-quantitative assessment of repair tissue. This score regions-of-interest (ROI) were also defined by the same radiologist on two or three consecutive slices on T_2_ mapping sequence (usually on third echo) images using JiveX (Visus, Bochum, Germany). The number of slices varied according to the extent of the repair tissue. For each slice, repair cartilage, cartilage adjacent to the repair cartilage, and a reference cartilage were defined (see **Fig. 1**). An adjacent ROI was always positioned next to the repair ROI. Reference ROI was placed at least 10 mm from the edge of the repair tissue. ROIs were positioned in the same area on the cartilage at all time-points. ROIs were then transferred onto corresponding slices of T_2_ maps for processing using MATLAB R2019.a (MathWorks, Natick, MA), as illustrated in **Fig. 1**. Mean T_2_ was calculated and averaged through the slices, ROI-wise, resulting in three T_2_ values per patient per time-point (one for repair cartilage, one for adjacent cartilage, and one for reference cartilage).

### GLCM Analysis

Only ROIs with more than 15 pixels were analyzed with GLCM. Using an in-house-written script in MATLAB, ROIs were rotated (built-in MATLAB functions “regionprops” and “imrotate”), flattened and quantized into 16 gray levels and consecutive GLCM analysis was computed with an offset of 0° (direction parallel to the cartilage surface) and a step of length 1 (considering a pixel and its immediate neighbor), as illustrated in **Fig. 1**. Using the *GLCM_features1* function from the MATLAB Repository,^33^ 20 quantitative features were extracted (*autocorrelation, cluster prominence, cluster shade, contrast, correlation, difference entropy, difference variance, dissimilarity, energy, entropy, homogeneity, information measure, information measure of correlation 2, inverse difference moment normalized, inverse difference normalized INN, maximum probability, sum average, sum entropy, sum of squares, sum variance*). For each patient at each time-point, GLCM features values were averaged through the slices, ROI-wise, resulting in nine values for every feature per patient (*repair cartilage* at 3M, 12M, and 24M; *adjacent cartilage* at 3M, 12M, and 24M; *reference cartilage* at 3M, 12M, and 24M). For visualization purposes, features heatmaps of repair tissue were calculated for one MACT and one MFX patient at each time-point. For rotated and flattened ROI, autocorrelation, homogeneity, and dissimilarity were calculated based on surrounding pixels selected by a 5 × 5-pixel sliding window with a step of length 1. These features were calculated in eight directions (0°, 45°, 90°, 135°, 180°, 225°, 270°, and 315°) and averaged, yielding three maps per patient per time-point. Map calculation and visualization was performed in Python 3.9.18.

### Statistical Analysis

All statistical analyses were performed using R version 4.2.2 (R Foundation for Statistical Computing, Vienna, Austria) in RStudio version 2022.07.2 (Rstudio, PBC). The Shapiro-Wilk test was used to assess the normality of the examined variables. Based on normality, Student’s *t* test or the Wilcoxon signed-rank test was used to determine the significance of differences in mean T_2_ values and GLCM features between the procedures, as well as all combinations of time-points for MACT and MFX separately. Differences are described as mean difference ± standard error of the difference between means. The Spearman correlation coefficient was used to examine the relationship between GLCM features and the MOCART 2.0 score. Based on simple comparisons between time-points, mean T_2_, autocorrelation, dissimilarity, homogeneity, correlation, sum average, and information measure were further analyzed. (See Appendix A, Supplemental Digital Content 1, which provides mathematical formulas and explanations of selected features.)

Linear mixed-effects models with a random slope and a random intercept with Kenward-Roger degrees of freedom correction were fitted using the function *lmer* from the R package *lme4 version 1.1-35.1*.^34^ In all models, we considered procedure, time-point and ROI type as fixed effects and we allowed individual intercepts for each patient with the slopes of the variable *ROI type* varying by patient. Final models were fitted with Restricted Maximum Likelihood (REML). We present the results as estimated differences with 95% confidence intervals. *P*-values lower than 0.05 were considered statistically significant.

## Results

### Correlation of GLCM Features with MOCART 2.0

Correlation analysis showed weak to strong correlation of GLCM features and the MOCART 2.0 score at different time-points. In the case of MACT, significant correlations were found between the MOCART 2.0 and autocorrelation (3M, *r* = −0.34), contrast (12M, *r* = −0.49; 24M, *r* = −0.58), correlation (12M, *r* = 0.35; 24M, *r* = 0.48), dissimilarity (12M, *r* = −0.47; 24M, *r* = −0.53), homogeneity (12M, *r* = 0.52; 24M, *r* = 0.44), sum of squares (3M, *r* = −0.36), difference variance (12M, *r* = −0.49; 24M, *r* = −0.58), difference entropy (12M, *r* = −0.43; 24M, *r* = −0.55), difference variance (12M, *r* = −0.49; 24M, *r* = −0.58), information measure (24M, *r* = −0.42), inverse difference normalized INN (12M, *r* = 0.51; 24M, *r* = 0.50), and inverse difference moment normalized (12M, *r* = 0.49; 24M, *r* = 0.58).

For MFX, the MOCART 2.0 score was significantly correlated with mean T_2_ (12M, *r* = −0.82), correlation (3M, *r* = 0.64), cluster shade (3M, *r* = 0.68; 24M, *r* = −0.61), sum average (3M, *r* = −0.68), sum entropy (12M, *r* = −0.69), and information measure of correlation 2 (3M, *r* = 0.69). All correlations are listed in **Table 1**.

**Table 1.** Correlations between MOCART 2.0 and Gray-Level Co-Occurrence Matrix (GLCM) Features.

	MACT			MFX		
	3M	12M	24M	3M	12M	24M
*T_2_*	–0.18	0.04	–0.09	–0.19	–0.82*	–0.15
Autocorrelation	–0.34*	–0.14	0.12	**–0.56**	0.18	**–0.31**
Contrast	–0.04	–0.49*	–0.58*	**–0.56**	**–0.37**	–0.20
Correlation	–0.05	0.35*	0.48*	0.64*	**–0.50**	**–0.37**
Cluster prominence	–0.06	–0.05	–0.10	**0.41**	**–0.37**	0.00
Cluster shade	0.15	0.13	–0.23	0.68*	–0.61*	0.06
Dissimilarity	–0.01	–0.47*	–0.53*	**–0.47**	–0.13	–0.10
Energy	–0.07	0.05	0.25	0.15	0.25	0.26
Entropy	0.01	–0.09	–0.19	–0.06	**–0.34**	–0.19
Homogeneity	–0.04	0.52*	0.44*	**0.47**	0.03	–0.03
Maximum probability	–0.11	–0.05	0.24	0.15	–0.08	0.05
Sum of squares	–0.36*	–0.14	0.12	**–0.56**	0.06	**–0.31**
Sum average	**–0.31**	–0.13	0.08	–0.68*	0.18	–0.20
Sum variance	**–0.32**	–0.17	0.11	**–0.56**	0.13	**–0.31**
Sum entropy	–0.07	0.16	0.06	**0.30**	–0.69*	**–0.46**
Difference variance	–0.04	–0.49*	–0.58*	**–0.56**	**–0.37**	–0.20
Difference entropy	–0.03	–0.43*	–0.55*	**–0.44**	–0.28	–0.20
Information measure	0.04	–0.24	–0.42*	**–0.56**	**0.39**	0.28
Information measure of correlation 2	–0.08	0.27	0.31	0.69*	**–0.50**	**–0.38**
Inverse difference normalized INN	–0.01	0.51*	0.50*	**0.47**	0.13	0.03
Inverse difference moment normalized	0.02	0.49*	0.58*	**0.56**	**0.37**	0.20

MACT = matrix-associated autologous chondrocyte transplantation; MFX = microfracturing; 3M = 3 months follow-up; 12M = 12 months follow-up; 24M = 24 months follow-up.

* Statistically significant correlations are marked with an asterisk. *P*-Values < 0.05 are Marked in Bold.

### Comparison of MACT and MFX

For a descriptive statistics of all parameters, see Appendix B, Supplemental Digital Content 1.

**Figure 2.**
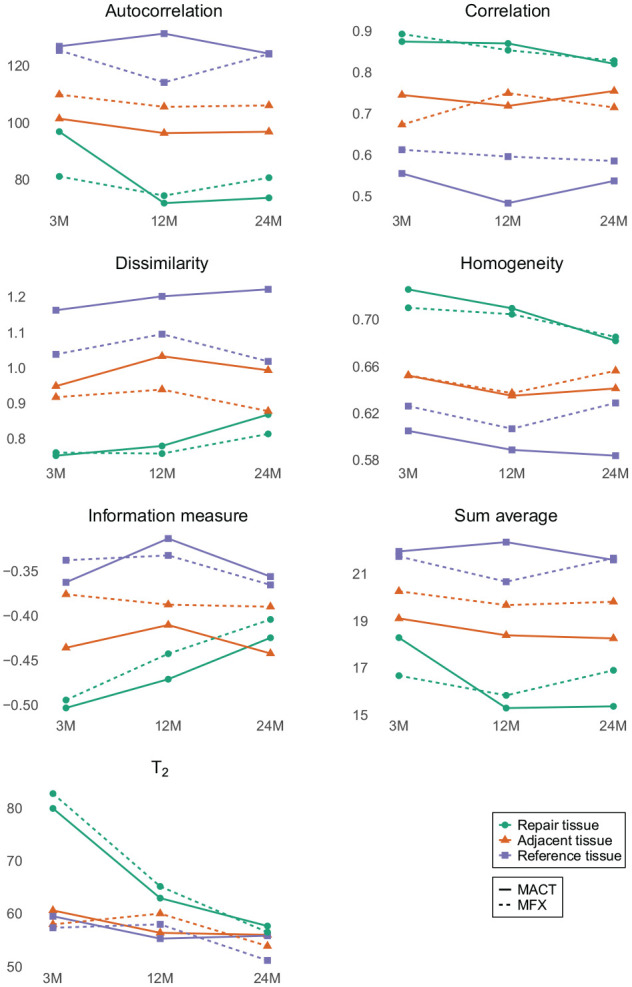
Mean values of the T_2_ and the selected gray-level co-occurrence matrix (GLCM) features at 3, 12, and 24 months after surgery.

Based on unpaired tests, we found no significant differences in the mean T_2_ or GLCM features between MACT and MFX across all time points for all regions of interest (ROIs), except for cluster prominence at 24 months in adjacent tissue (*P* = 0.034). When examining the changes in these parameters separately for each procedure, it was observed that, for MFX, only the mean T_2_ showed significant differences, specifically between 3M and 12M (*P* < 0.001) and between 3M and 24M (*P* = 0.004). For MACT, mean T_2_ values also changed significantly between 3M and 12M (*P* < 0.001), 3M and 24M (*P* < 0.001), and between 12M and 24M (*P* = 0.034). Furthermore, GLCM features changed significantly for MACT, namely, autocorrelation, correlation, homogeneity, information measure, information measure of correlation 2, sum average, sum of squares, and sum variance. All significant differences are listed in **Table 2**. The variables chosen for further analysis via linear mixed-effects models are visualized in **Fig. 2.**

**Table 2.** Significant Differences in Gray-Level Co-Occurrence Matrix (GLCM) Features between Time-Points.

ROI	Procedure	Parameter	Contrast	Mean difference	SE	*P*-value
Repair tissue	MACT	Autocorrelation	3M vs. 12M	25.11	9.29	0.001
Repair tissue	MACT	Autocorrelation	3M vs. 24M	23.25	9.93	0.014
Repair tissue	MACT	Correlation	12M vs. 24M	0.05	0.03	0.034
Repair tissue	MACT	Homogeneity	12M vs. 24M	0.03	0.02	0.049
Repair tissue	MACT	Information measure	3M vs. 24M	–0.08	0.03	0.016
Repair tissue	MACT	Information measure of correlation 2	3M vs. 24M	0.04	0.02	0.031
Repair tissue	MACT	Information measure of correlation 2	12M vs.24M	0.03	0.02	0.031
Repair tissue	MACT	Sum average	3M vs. 12M	2.99	1.03	0.001
Repair tissue	MACT	Sum average	3M vs. 24M	2.91	1.14	0.012
Repair tissue	MACT	Sum of squares	3M vs. 12M	24.9	9.25	0.001
Repair tissue	MACT	Sum of squares	3M vs. 24M	22.98	9.88	0.013
Repair tissue	MACT	Sum variance	3M vs. 12M	88.85	35.41	0.001
Repair tissue	MACT	Sum variance	3M vs. 24M	80.36	36.82	0.015
Repair tissue	MACT	Mean T_2_	3M vs. 12M	16.98	4.94	0.000
Repair tissue	MACT	Mean T_2_	3M vs. 24M	22.27	5.18	0.000
Repair tissue	MACT	Mean T_2_	12M vs. 24M	5.28	4.26	0.034
Repair tissue	MFX	Mean T_2_	3M vs. 12M	17.58	4.76	0.012
Repair tissue	MFX	Mean T_2_	3M vs. 24M	26.2	6.36	0.012
Adjacent issue	MACT	Mean T_2_	3M vs. 24M	4.63	4.54	0.042
Reference tissue	MACT	Energy	3M vs. 12M	0.02	0.01	0.016
Reference tissue	MACT	Information measure	3M vs. 12M	-0.05	0.03	0.045
Reference tissue	MACT	Information measure	12M vs. 24M	0.04	0.03	0.045
Reference tissue	MACT	Maximum probability	3M vs. 12M	0.03	0.01	0.04

* MACT = matrix-associated autologous chondrocyte transplantation; MFX = microfracturing; 3M = 3 months follow-up; 12M = 12 months follow-up; 24M = 24 months follow-up.

### Linear Mixed-Effects Models

Models did not show significant differences between the procedures (*P* > 0.05). However, when analyzing MACT and MFX separately, we found a significant decrease in the T_2_ of repair tissue in the MACT group (3Mvs12M: *P* < 0.001; 3Mvs24M: *P* < 0.001) and in the MFX group (3Mvs12M: *P* = 0.001; 3Mvs24M: *P* < 0.001; 12Mvs24M: *P* = 0.034).

Moreover, there was a significant change in autocorrelation (3Mvs12M: *P* = 0.002; 3Mvs24M: *P* = 0.004), dissimilarity (3Mvs24M: *P* = 0.01), homogeneity (3Mvs24M: *P* = 0.013), correlation (3Mvs24M: *P* = 0.036), sum average (3Mvs12M: *P* = 0.001; 3Mvs24M: *P* = 0.002) and information measure (3Mvs24M: *P* < 0.001) in the MACT repair tissue. The only significant change in the texture of MFX was observed in the repair tissue, as measured by the information measure (3M vs 24M: *P* < 0.016). All estimated differences in time are provided in **Table 3**.

**Table 3.** Differences between Time-Points in All Regions of Interest (ROIs) Estimated by the Models. In the Case of Logarithmic Variable Transformation During Modeling, the Difference Is Expressed as a Ratio in the Original Scale.

Fixed variable: Tissue type	Variable contrasts*	T_2_ (ms)(ratio)	95% CI			*p*-value	Autocorrelation(difference)	95% CI			*p*-value	Dissimilarity(ratio)	95% CI			*p*-value
MACT																
Repair tissue																
	3M vs. 12M	1.26	1.14	–	1.39	**<0.001**	25.1	8.04	–	42.16	**0.002**	0.93	0.80	–	1.08	0.501
	3M vs. 24M	1.39	1.25	–	1.54	**<0.001**	23.25	6.18	–	40.31	**0.004**	0.83	0.71	–	0.96	**0.010**
	12M vs. 24M	1.1	1	–	1.22	0.063	–1.86	–18.92	–	15.21	0.964	0.89	0.77	–	1.03	0.162
Adjacent tissue																
	3M vs. 12M	1.06	0.96	–	1.18	0.344	5.07	–11.99	–	22.13	0.763	0.93	0.80	–	1.08	0.511
	3M vs. 24M	1.07	0.97	–	1.19	0.231	4.6	–12.47	–	21.66	0.801	0.93	0.80	–	1.09	0.537
	12M vs. 24M	1.01	0.91	–	1.12	0.968	–0.47	–17.54	–	16.59	0.998	1.00	0.86	–	1.16	0.999
Reference tissue																
	3M vs. 12M	1.06	0.96	–	1.18	0.334	–4.46	–21.52	–	12.6	0.811	0.95	0.82	–	1.11	0.711
	3M vs. 24M	1.06	0.95	–	1.17	0.412	2.56	–14.5	–	19.62	0.933	0.92	0.80	–	1.07	0.434
	12M vs. 24M	0.99	0.9	–	1.1	0.989	7.02	–10.04	–	24.08	0.596	0.97	0.84	–	1.13	0.896
**MFX**																
Repair tissue																
	3M vs. 12M	1.28	1.08	–	1.51	**0.002**	6.74	–21.29	–	34.78	0.837	0.99	0.78	–	1.27	0.999
	3M vs. 24M	1.53	1.29	–	1.81	**<0.001**	0.44	–27.6	–	28.47	0.999	0.91	0.71	–	1.16	0.622
	12M vs. 24M	1.2	1.01	–	1.41	**0.034**	–6.3	–34.34	–	21.73	0.856	0.91	0.71	–	1.17	0.654
Adjacent tissue																
	3M vs. 12M	0.96	0.82	–	1.14	0.865	4.27	–23.76	–	32.31	0.931	0.95	0.74	–	1.22	0.887
	3M vs. 24M	1.1	0.93	–	1.31	0.342	3.79	–24.25	–	31.82	0.945	1.05	0.82	–	1.34	0.884
	12M vs. 24M	1.15	0.97	–	1.36	0.137	–0.48	–28.52	–	27.55	0.999	1.10	0.86	–	1.41	0.616
Reference tissue																
	3M vs. 12M	0.99	0.84	–	1.17	0.995	11.24	–16.79	–	39.28	0.611	0.89	0.69	–	1.14	0.499
	3M vs. 24M	1.14	0.96	–	1.35	0.168	1.27	–26.76	–	29.31	0.994	0.99	0.77	–	1.27	0.996
	12M vs. 24M	1.15	0.97	–	1.36	0.138	–9.97	–38	–	18.07	0.679	1.12	0.87	–	1.43	0.550
		Homogeneity(difference)	95% CI			*p*-value	Correlation(difference)	95% CI			*p*-value	Sum average(difference)	95% CI			*p*-value
**MACT**																
Repair tissue																
	3M vs. 12M	0.02	–0.02	–	0.05	0.545	0.01	–0.08	–	0.11	0.919	2.99	1.01	–	4.96	**0.001**
	3M vs. 24M	0.04	0.01	–	0.08	**0.013**	0.10	0.01	–	0.19	**0.019**	2.91	0.94	–	4.89	**0.002**
	12M vs. 24M	0.03	–0.01	–	0.06	0.168	0.09	0.00	–	0.18	0.053	–0.07	–2.05	–	1.90	0.996
Adjacent tissue																
	3M vs. 12M	0.02	–0.02	–	0.05	0.5	0.04	–0.05	–	0.13	0.545	0.72	–1.26	–	2.69	0.669
	3M vs. 24M	0.01	–0.03	–	0.05	0.755	–0.01	–0.10	–	0.08	0.925	0.85	–1.13	–	2.82	0.570
	12M vs. 24M	–0.01	–0.04	–	0.03	0.912	–0.05	–0.15	–	0.04	0.328	0.13	–1.84	–	2.11	0.986
Reference tissue																
	3M vs. 12M	0.02	–0.02	–	0.05	0.54	0.04	–0.05	–	0.13	0.549	–0.39	–2.37	–	1.58	0.885
	3M vs. 24M	0.02	–0.01	–	0.06	0.352	–0.01	–0.10	–	0.08	0.937	0.36	–1.62	–	2.33	0.903
	12M vs. 24M	0	–0.03	–	0.04	0.944	–0.05	–0.14	–	0.04	0.349	0.75	–1.22	–	2.73	0.640
**MFX**																
Repair tissue																
	3M vs. 12M	0.01	–0.05	–	0.06	0.974	0.08	–0.07	–	0.23	0.401	0.84	–2.41	–	4.08	0.816
	3M vs. 24M	0.02	–0.03	–	0.08	0.584	0.13	–0.01	–	0.28	0.085	–0.23	–3.47	–	3.02	0.985
	12M vs. 24M	0.02	–0.04	–	0.08	0.722	0.05	–0.10	–	0.20	0.676	–1.06	–4.31	–	2.18	0.720
Adjacent tissue																
	3M vs. 12M	0.02	–0.04	–	0.07	0.819	–0.09	–0.24	–	0.06	0.336	0.59	–2.66	–	3.83	0.905
	3M vs. 24M	0	–0.06	–	0.06	0.987	–0.05	–0.20	–	0.10	0.675	0.45	–2.80	–	3.69	0.944
	12M vs. 24M	–0.02	–0.08	–	0.04	0.729	0.04	–0.11	–	0.18	0.838	–0.14	–3.39	–	3.10	0.994
Reference tissue																
	3M vs. 12M	0.02	–0.04	–	0.08	0.723	–0.01	–0.16	–	0.14	0.990	1.07	–2.17	–	4.32	0.716
	3M vs. 24M	0	–0.06	–	0.06	0.994	0.03	–0.12	–	0.17	0.913	0.07	–3.17	–	3.32	0.998
	12M vs. 24M	–0.02	–0.08	–	0.04	0.657	0.03	–0.11	–	0.18	0.853	–1.00	–4.25	–	2.25	0.748
		Information measure(difference)	95% CI			*p*-value										
**MACT**																
Repair tissue																
	3M vs. 12M	–0.03	–0.08	–	0.01	0.237										
	3M vs. 24M	–0.08	–0.13	–	–0.03	**<0.001**										
	12M vs. 24M	–0.05	–0.09	–	0.00	0.051										
Adjacent tissue																
	3M vs. 12M	–0.03	–0.07	–	0.02	0.402										
	3M vs. 24M	0.01	–0.04	–	0.05	0.944										
	12M vs. 24M	0.03	–0.01	–	0.08	0.243										
Reference tissue																
	3M vs. 12M	–0.05	–0.10	–	0.00	0.037										
	3M vs. 24M	–0.01	–0.05	–	0.04	0.943										
	12M vs. 24M	0.04	0.00	–	0.09	0.081										
**MFX**																
Repair tissue																
	3M vs. 12M	–0.05	–0.13	–	0.02	0.249										
	3M vs. 24M	–0.09	–0.17	–	–0.01	**0.016**										
	12M vs. 24M	–0.04	–0.12	–	0.04	0.465										
Adjacent tissue																
	3M vs. 12M	0.01	–0.07	–	0.09	0.933										
	3M vs. 24M	0.01	–0.06	–	0.09	0.904										
	12M vs. 24M	0.00	–0.07	–	0.08	0.997										
Reference tissue																
	3M vs. 12M	–0.01	–0.08	–	0.07	0.985										
	3M vs. 24M	0.03	–0.05	–	0.10	0.671										
	12M vs. 24M	0.03	–0.04	–	0.11	0.566										

MACT = matrix-associated autologous chondrocyte transplantation; MFX = microfracturing; 3M = 3 months follow-up; 12M = 12 months follow-up; 24M = 24 months follow-up. *P*-Values < 0.05 are Marked in Bold.

Mean T_2_ differed significantly between repair and adjacent tissue and repair and reference tissue in both MACT and MFX (all *P* < 0.001). In MACT, models revealed differences in GLCM between all combinations of ROI types at almost all time-points (see **Table 4**). In the case of MFX, the significant differences were mainly between repair and reference tissue at 12 months. For all comparisons, please refer to **Table 4**.

**Table 4.** Differences Between Regions of Interest (ROIs) at All Time-Points Estimated by the Models. In Case of Logarithmic Variable Transformation During Modeling, the Difference Is Expressed as a Ratio in the Original Scale.

Fixed variable: Tissue type	Variable contrasts*	T_2_ (ms)(ratio)	95% CI			*p*-value	Autocorrelation(difference)	95% CI			*p*-value	Dissimilarity(ratio)	95% CI			*p*-value
MACT																
3M																
	repair vs. adjacent	1.32	1.16	–	1.51	**<0.001**	–4.55	–26.56	–	17.45	0.875	0.78	0.64	–	0.94	**0.005**
	repair vs. reference	1.33	1.16	–	1.53	**<0.001**	–29.96	–50.34	–	–9.58	**0.002**	0.64	0.52	–	0.78	**<0.001**
	adjacent vs. reference	1.01	0.9	–	1.12	0.991	–25.4	–44.41	–	–6.39	**0.005**	0.82	0.70	–	0.96	**0.008**
12M																
	repair vs. adjacent	1.12	0.98	–	1.28	0.122	–24.59	–46.59	–	–2.58	**0.025**	0.78	0.64	–	0.94	**0.005**
	repair vs. reference	1.13	0.98	–	1.29	0.107	–59.52	–79.9	–	–39.14	**<0.001**	0.65	0.53	–	0.80	**<0.001**
	adjacent vs. reference	1.01	0.9	–	1.13	0.988	–34.94	–53.95	–	–15.93	**<0.001**	0.84	0.71	–	0.98	**0.021**
24M																
	repair vs. adjacent	1.02	0.9	–	1.17	0.906	–23.21	–45.21	–	–1.2	**0.036**	0.88	0.72	–	1.06	0.280
	repair vs. reference	1.01	0.88	–	1.16	0.969	–50.65	–71.03	–	–30.27	**<0.001**	0.71	0.58	–	0.87	**<0.001**
	adjacent vs. reference	0.99	0.89	–	1.11	0.976	–27.44	–46.45	–	–8.43	**0.002**	0.81	0.69	–	0.95	**0.005**
MFX																
3M																
	repair vs. adjacent	1.44	1.16	–	1.8	**<0.001**	–28.71	–64.87	–	7.44	0.147	0.82	0.60	–	1.12	0.382
	repair vs. reference	1.46	1.17	–	1.83	**<0.001**	–44.26	–77.75	–	–10.77	**0.006**	0.76	0.54	–	1.06	0.145
	adjacent vs. reference	1.01	0.85	–	1.22	0.981	–15.55	–46.78	–	15.69	0.467	0.92	0.71	–	1.19	1.000
12M																
	repair vs. adjacent	1.09	0.87	–	1.35	0.646	–31.19	–67.34	–	4.97	0.105	0.79	0.58	–	1.07	0.191
	repair vs. reference	1.13	0.9	–	1.42	0.382	–39.76	–73.25	–	–6.27	**0.016**	0.68	0.48	–	0.95	**0.018**
	adjacent vs. reference	1.05	0.87	–	1.25	0.835	–8.57	–39.81	–	22.66	0.792	0.86	0.66	–	1.12	0.488
24M																
	repair vs. adjacent	1.04	0.84	–	1.3	0.902	–25.36	–61.52	–	10.79	0.222	0.95	0.70	–	1.30	1.000
	repair vs. reference	1.09	0.87	–	1.36	0.653	–43.42	–76.91	–	–9.93	**0.007**	0.83	0.59	–	1.16	0.524
	adjacent vs. reference	1.04	0.87	–	1.25	0.837	–18.06	–49.3	–	13.18	0.359	0.87	0.67	–	1.13	0.585
		Homogeneity(difference)	95% CI			*p*-value	Correlation(difference)	95% CI			*p*-value	Sum average(difference)	95% CI			*p*-value
MACT																
3M																
	repair vs. adjacent	0.07	0.03	–	0.12	**<0.001**	0.22	0.09	–	0.35	**<0.001**	–0.82	–3.40	–	1.76	0.732
	repair vs. reference	0.12	0.07	–	0.17	**<0.001**	0.41	0.25	–	0.56	**<0.001**	–3.66	–6.08	–	–1.24	**0.001**
	adjacent vs. reference	0.05	0.01	–	0.09	**0.012**	0.19	0.09	–	0.29	**<0.001**	–2.84	–5.05	–	–0.64	**0.008**
12M																
	repair vs. adjacent	0.07	0.03	–	0.12	**<0.001**	0.24	0.11	–	0.37	**<0.001**	–3.09	–5.67	–	–0.51	**0.015**
	repair vs. reference	0.12	0.07	–	0.17	**<0.001**	0.43	0.28	–	0.58	**<0.001**	–7.04	–9.46	–	–4.62	**<0.001**
	adjacent vs. reference	0.05	0.01	–	0.08	**0.014**	0.19	0.09	–	0.29	**<0.001**	–3.95	–6.16	–	–1.75	**0.000**
24M																
	repair vs. adjacent	0.04	–0.01	–	0.09	0.101	0.10	–0.03	–	0.23	0.183	–2.88	–5.46	–	–0.30	**0.025**
	repair vs. reference	0.1	0.05	–	0.15	**<0.001**	0.29	0.14	–	0.44	**<0.001**	–6.21	–8.63	–	–3.79	**0.000**
	adjacent vs. reference	0.06	0.02	–	0.1	**0.002**	0.19	0.09	–	0.29	**<0.001**	–3.33	–5.53	–	–1.12	**0.001**
**MFX**																
3M																
	repair vs. adjacent	0.06	–0.02	–	0.13	0.178	0.34	0.13	–	0.56	**0.001**	–3.58	–7.82	–	0.66	0.115
	repair vs. reference	0.08	0	–	0.17	0.057	0.42	0.17	–	0.67	**<0.001**	–5.06	–9.03	–	–1.08	**0.009**
	adjacent vs. reference	0.03	–0.04	–	0.09	0.591	0.08	–0.09	–	0.25	0.497	–1.48	–5.10	–	2.15	0.599
12M																
	repair vs. adjacent	0.07	–0.01	–	0.14	0.097	0.17	–0.04	–	0.39	0.137	–3.83	–8.07	–	0.41	0.085
	repair vs. reference	0.1	0.01	–	0.18	**0.022**	0.33	0.08	–	0.58	**0.006**	–4.82	–8.80	–	–0.84	**0.013**
	adjacent vs. reference	0.03	–0.03	–	0.09	0.494	0.16	–0.01	–	0.33	0.064	–0.99	–4.61	–	2.63	0.794
24M																
	repair vs. adjacent	0.03	–0.05	–	0.11	0.644	0.16	–0.06	–	0.37	0.198	–2.91	–7.15	–	1.33	0.237
	repair vs. reference	0.06	–0.03	–	0.14	0.266	0.31	0.06	–	0.56	0.011	–4.76	–8.73	–	–0.78	**0.015**
	adjacent vs. reference	0.03	–0.04	–	0.09	0.561	0.16	–0.01	–	0.33	0.068	–1.85	–5.47	–	1.77	0.449
		Information measure(difference)	95% CI			*p*-value										
**MACT**																
3M																
	repair vs. adjacent	–0.07	–0.13	–	0.00	**0.037**										
	repair vs. reference	–0.14	–0.22	–	–0.06	**<0.001**										
	adjacent vs. reference	–0.07	–0.13	–	–0.02	**0.008**										
12M																
	repair vs. adjacent	–0.06	–0.12	–	0.00	**0.067**										
	repair vs. reference	–0.16	–0.23	–	–0.08	**<0.001**										
	adjacent vs. reference	–0.10	–0.15	–	–0.04	**<0.001**										
24M																
	repair vs. adjacent	0.02	–0.05	–	0.08	0.790										
	repair vs. reference	–0.07	–0.15	–	0.01	0.089										
	adjacent vs. reference	–0.09	–0.14	–	–0.03	**0.002**										
**MFX**																
3M																
	repair vs. adjacent	0.02	–0.22	–	–0.01	**0.023**										
	repair vs. reference	–0.07	–0.28	–	–0.03	**0.011**										
	adjacent vs. reference	–0.09	–0.13	–	0.06	0.600										
12M																
	repair vs. adjacent	–0.05	–0.16	–	0.05	0.429										
	repair vs. reference	–0.11	–0.24	–	0.02	0.098										
	adjacent vs. reference	–0.06	–0.15	–	0.04	0.348										
24M																
	repair vs. adjacent	–0.01	–0.12	–	0.09	0.944										
	repair vs. reference	–0.04	–0.16	–	0.09	0.742										
	adjacent vs. reference	–0.02	–0.12	–	0.07	0.811										

MACT = matrix-associated autologous chondrocyte transplantation; MFX = microfracturing; 3M = 3 months follow-up; 12M = 12 months follow-up; 24M = 24 months follow-up. *P*-Values < 0.05 are Marked in Bold.

We found a significant effect of time-point 24M in all models: T_2_ (*P* < 0.001); autocorrelation (*P* = 0.002); dissimilarity (*P* = 0.003); homogeneity (*P* = 0.005); correlation (*P* = 0.007); sum average (*P* = 0.001); and information measure (*P* < 0.001). The effect of reference tissue was also significant in all models: T_2_ (*P* < 0.001); autocorrelation (*P* = 0.001); dissimilarity (*P* < 0.001); homogeneity (*P* < 0.001); correlation (*P* < 0.001); sum average (*P* = 0.001); and information measure (*P* < 0.001). All significant effects and interactions are listed in **Table 5**.

**Table 5.** Summary of Linear Mixed Effects Models of T_2_ and Selected Gray-Level Co-Occurrence Matrix (GLCM) Features in Repair, Adjacent, and Reference Tissue at 3, 12, and 24 Months after Surgery. To Achieve Adequate Model Fit, It Was Necessary to Apply a Natural Logarithm Transformation to Both T_2_ and Dissimilarity Variables and a Cubic Transformation to the Correlation Variable.

Predictors	Log (mean T_2_)			Autocorrelation			log(Dissimilarity)			Homogeneity			Correlation^3^			Sum average			Information measure		
	Estimates	CI	*P*	Estimates	CI	*p*	Estimates	CI	*p*	Estimates	CI	*p*	Estimates	CI	*p*	Estimates	CI	*P*	Estimates	CI	*p*
(Intercept)	4.35	4.26 to 4.44	**<0.001**	96.79	84.14 to 109.45	**<0.001**	−0.4	−0.56 to −0.24	**<0.001**	0.73	0.69 to 0.76	**<0.001**	0.69	0.62 to 0.77	**<0.001**	18.28	16.82 to 19.74	**<0.001**	−0.5	−0.54 to −0.46	**<0.001**
Time-point 12M	−0.23	−0.31 to −0.14	**<0.001**	−25.1	−39.35 to −10.85	**0.001**	0.07	−0.05 to 0.20	0.263	−0.02	−0.05 to 0.01	0.294	−0.01	−0.09 to 0.06	0.696	−2.99	−4.64 to −1.34	**<0.001**	0.03	−0.01 to 0.07	0.106
Time-point 24M	−0.33	−0.41 to −0.24	**<0.001**	−23.25	−37.50 to −9.00	**0.002**	0.19	0.06 to 0.31	**0.003**	−0.04	−0.07 to −0.01	**0.005**	−0.1	−0.18 to −0.03	**0.007**	−2.91	−4.56 to −1.26	**0.001**	0.08	0.04 to 0.12	**<0.001**
Adjacent tissue	−0.28	−0.39 to −0.17	**<0.001**	4.55	−13.79 to 22.90	0.623	0.25	0.10 to 0.41	**0.002**	−0.07	−0.11 to −0.03	**<0.001**	−0.22	−0.32 to −0.11	**<0.001**	0.82	−1.33 to 2.97	0.453	0.07	0.01 to 0.12	**0.014**
Reference tissue	−0.29	−0.40 to −0.17	**<0.001**	29.96	12.96 to 46.96	**0.001**	0.45	0.28 to 0.62	**<0.001**	−0.12	−0.16 to −0.08	**<0.001**	−0.41	−0.53 to −0.28	**<0.001**	3.66	1.64 to 5.68	**0.001**	0.14	0.08 to 0.20	**<0.001**
Procedure MFX	0.06	−0.11 to 0.24	0.478	−15.73	−40.07 to 8.60	0.203	0.06	−0.25 to 0.36	0.715	−0.02	−0.08 to 0.05	0.634	0.03	−0.12 to 0.18	0.694	−1.61	−4.42 to 1.19	0.257	0.01	−0.07 to 0.09	0.823
Time-point 12M * Adjacent tissue	0.17	0.05 to 0.29	**0.006**	20.03	−0.12 to 40.18	0.051	0	−0.18 to 0.18	0.991	0	−0.04 to 0.04	0.959	−0.03	−0.13 to 0.08	0.64	2.27	−0.06 to 4.60	0.056	−0.01	−0.06 to 0.05	0.813
Time-point 24M * Adjacent tissue	0.26	0.14 to 0.38	**<0.001**	18.65	−1.50 to 38.80	0.069	−0.12	−0.30 to 0.06	0.183	0.03	−0.01 to 0.08	0.13	0.12	0.01 to 0.23	**0.029**	2.07	−0.27 to 4.40	0.082	−0.08	−0.14 to −0.03	**0.003**
Time-point 12M * Reference tissue	0.17	0.05 to 0.29	**0.007**	29.56	9.41 to 49.72	**0.004**	−0.02	−0.20 to 0.16	0.813	0	−0.04 to 0.04	0.996	−0.03	−0.13 to 0.08	0.644	3.38	1.05 to 5.71	**0.005**	0.02	−0.04 to 0.07	0.546
Time-point 24M * Reference tissue	0.27	0.15 to 0.39	**<0.001**	20.69	0.54 to 40.84	**0.044**	−0.11	−0.29 to 0.07	0.225	0.02	−0.02 to 0.07	0.296	0.12	0.01 to 0.22	**0.031**	2.55	0.22 to 4.89	**0.032**	−0.07	−0.13 to −0.02	**0.011**
Time-point 12M * MFX	−0.02	−0.18 to 0.15	0.835	18.36	−9.05 to 45.77	0.188	−0.07	−0.31 to 0.17	0.59	0.01	−0.05 to 0.07	0.719	−0.07	−0.21 to 0.08	0.369	2.15	−1.02 to 5.32	0.183	0.02	−0.06 to 0.09	0.604
Time-point 24M * MFX	−0.1	−0.26 to 0.07	0.242	22.81	−4.60 to 50.22	0.102	−0.09	−0.33 to 0.15	0.459	0.02	−0.04 to 0.08	0.52	−0.03	−0.18 to 0.12	0.685	3.14	−0.03 to 6.31	0.052	0.01	−0.06 to 0.09	0.759
Adjacent tissue * MFX	−0.09	−0.30 to 0.13	0.431	24.16	−11.13 to 59.45	0.177	−0.06	−0.35 to 0.24	0.714	0.02	−0.06 to 0.09	0.674	−0.13	−0.34 to 0.08	0.228	2.76	−1.37 to 6.90	0.188	0.05	−0.05 to 0.15	0.326
Reference tissue * MFX	−0.09	−0.31 to 0.13	0.401	14.3	−18.40 to 47.00	0.388	−0.17	−0.50 to 0.15	0.295	0.04	−0.05 to 0.12	0.385	−0.02	−0.26 to 0.23	0.89	1.4	−2.48 to 5.28	0.476	0.02	−0.11 to 0.14	0.796
Time-point 12M * Adjacent tissue * MFX	0.11	−0.12 to 0.35	0.333	−17.56	−56.32 to 21.20	0.373	0.04	−0.30 to 0.38	0.797	−0.01	−0.09 to 0.07	0.838	0.2	−0.01 to 0.40	0.062	−2.02	−6.51 to 2.46	0.375	−0.06	−0.16 to 0.05	0.292
Time-point 24M * Adjacent tissue * MFX	0.07	−0.16 to 0.30	0.558	−22	−60.76 to 16.76	0.264	−0.03	−0.37 to 0.31	0.879	0	−0.09 to 0.08	0.922	0.07	−0.14 to 0.27	0.51	−2.74	−7.23 to 1.75	0.23	−0.02	−0.13 to 0.09	0.722
Time-point 12M * Reference tissue * MFX	0.09	−0.15 to 0.32	0.468	−34.06	−72.83 to 4.70	0.085	0.13	−0.21 to 0.47	0.441	−0.01	−0.10 to 0.07	0.743	0.11	−0.09 to 0.32	0.272	−3.62	−8.11 to 0.87	0.113	−0.06	−0.17 to 0.04	0.24
Time-point 24M * Reference tissue * MFX	0.02	−0.21 to 0.26	0.839	−21.52	−60.29 to 17.24	0.275	0.02	−0.32 to 0.36	0.904	0	−0.08 to 0.09	0.907	−0.01	−0.21 to 0.20	0.934	−2.85	−7.34 to 1.63	0.211	−0.05	−0.15 to 0.06	0.395
Random Effects																					
σ^2^	0.03			705.33			0.05			0			0.02			9.45			0.01		
τ00 Patient	0.03			392.07			0.11			0			0.02			5.15			0.01		
τ11 Patient—Adjacent tissue	0.03			893.32			0.06			0			0.04			12.73			0.01		
τ11 Patient—Reference tissue	0.04			576.9			0.08			0.01			0.07			9.07			0.02		
ρ01	–0.39			–0.64			–0.16			–0.12			–0.05			–0.63			–0.58		
	–0.74			–0.49			–0.36			–0.37			–0.39			–0.59			–0.78		
ICC	0.54			0.4			0.7			0.67			0.7			0.4			0.55		
N Patient	37			37			37			37			37			37			37		
Observations	333			333			333			333			333			333			333		
Marginal R^2^ / Conditional R^2^	0.198 / 0.630			0.251 / 0.553			0.129 / 0.741			0.173 / 0.729			0.267 / 0.783			0.264 / 0.559			0.205 / 0.645		

MACT = matrix-associated autologous chondrocyte transplantation; MFX = microfracturing; 3M = 3 months follow-up; 12M = 12 months follow-up; 24M = 24 months follow-up. *P*-Values < 0.05 are Marked in Bold.

## Discussion

Inventing noninvasive, clinically relevant approaches for the assessment of cartilage repair tissue remains a persistent challenge. GLCM analysis was previously applied to monitoring of OA,^19-27,28^ but it is essential to recognize that the maturation of repair tissue is a different, yet similar process compared with cartilage degradation. As such, findings from OA studies are not directly transferable to our current investigation. Up to date published studies compared GLCM features of MACT and MFX at 24M after surgery^35^ and the interconnection between repair and adjacent tissue, suggesting that cartilage tissue adjacent to the repair site changes along with the cartilage implant.^36^ Therefore, we included both adjacent and reference tissue in this analysis. Although the T_2_ value is linked to the cartilage matrix structure, measured voxels are too large to distinguish fiber orientation precisely, T_2_ maps offer rough information about cartilage composition. Consequently, GLCM should be viewed not as an ultra-structural assessment, but as a metric between micro- and macroscopic.

This is the first study to analyze the correlation of textural features with the MOCART 2.0 score. Both surgical procedures show significant correlations with the MOCART 2.0 score at 3M and 12M. However, only textural features of MACT showed significant correlations with the MOCART score at 24M. Specifically, these were contrast (*r* = −0.58), correlation (*r* = 0.48), dissimilarity (*r* = −0.53), homogeneity (*r* = 0.44), difference variance (*r* = −0.58), difference entropy (= −0.55), difference variance (*r* = −0.58), information measure (24M, *r* = −0.42), inverse difference normalized INN (*r* = 0.50), and inverse difference moment normalized (*r* = 0.58). This suggests that GLCM texture of the repair tissue is tied to the morphological outcome and this relationship is more prominent in the MACT group mainly at the later follow-ups. As morphological change is preceded by micro-structural alteration^37^ and reflected by T_2_ values to some extent, a connection between textural features and the MOCART 2.0 score is logical. Experiments on cadaveric knees,^38^ demonstrated a correlation between the features autocorrelation, contrast, and entropy extracted from T_2_ maps with the histologically assessed stage of cartilage degeneration (Mankin score). Also, correlation between collagen fiber parallelism and feature autocorrelation was discovered. Schagemann *et al.*^39^ showed mild but significant correlation between the 3D MOCART score category *defect overall* and histological ICRS II *overall assessment* in a large-animal model at 3T. Goebel *et al.*^40^ found correlations between “defect fill” and “total points” of the 2D and 3D MOCART scores and the corresponding categories of the Sellers and Wakitani histological scores at 9.4T in an animal model. To date, we are not aware of any study investigating a direct relationship between MACT graft texture and histology; however, our results indicate a possible link.

Unpaired tests and linear mixed-effects models found no significant differences between MACT and MFX regarding T_2_ and texture, except for cluster prominence at 24M in adjacent tissue (*P* = 0.034), which was, potentially, a fluke among the multiple parameters tested. Previous studies demonstrated the ability of T_2_ mapping to differentiate between repair types;^29,41^ for example, Welsch *et al.*^29^ found significantly lower T_2_ values in MFX (47.9 ± 9.8 ms) compared with T_2_ values in MACT (53.6 ± 11.9 ms), which they linked to the fibrocartilaginous-like structure of MFX and the hyaline-like structure of MACT. In our cohort, changes in bulk T_2_ might not have been present at 24 months but might have developed later. A previous analysis,^35^ specifically focusing on femoral cartilage at the 24-month mark with a sample size of 57 patients, found significant differences in texture between MACT and MFX. These differences were observed in autocorrelation (*P* < 0.001), sum of squares (*P* < 0.001), sum average (*P* = 0.01), sum variance (*P* < 0.001), and sum entropy (*P* = 0.05). However, in the current analysis, a smaller subset of only 34 patients with measurements available at all three time-points was considered. Consequently, we were unable to demonstrate the differences between MACT and MFX in this subset.

**Figure 3.**
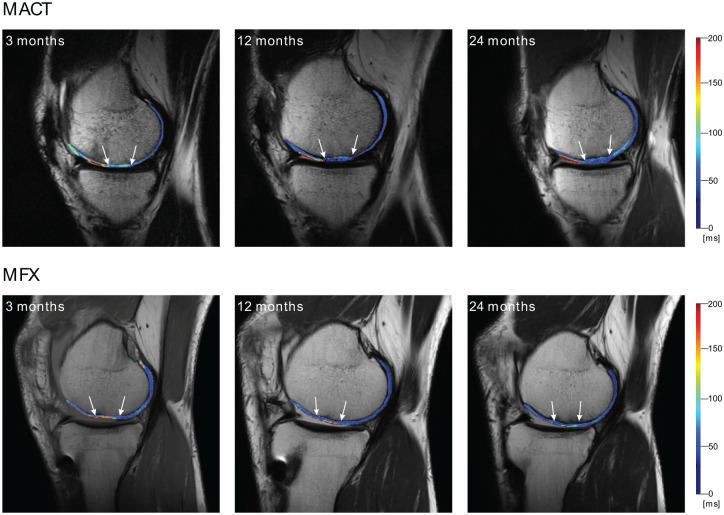
Example of cartilage repair healing. Examined tissue is delineated by white arrows. Matrix-associated autologous chondrocyte transplantation (MACT) change in T_2_ distribution is clearly visible between the 3M and 12M mark. Microfracturing (MFX) T_2_ distribution remains approximately the same, but the overall T_2_ values decrease.

**Figure 4.**
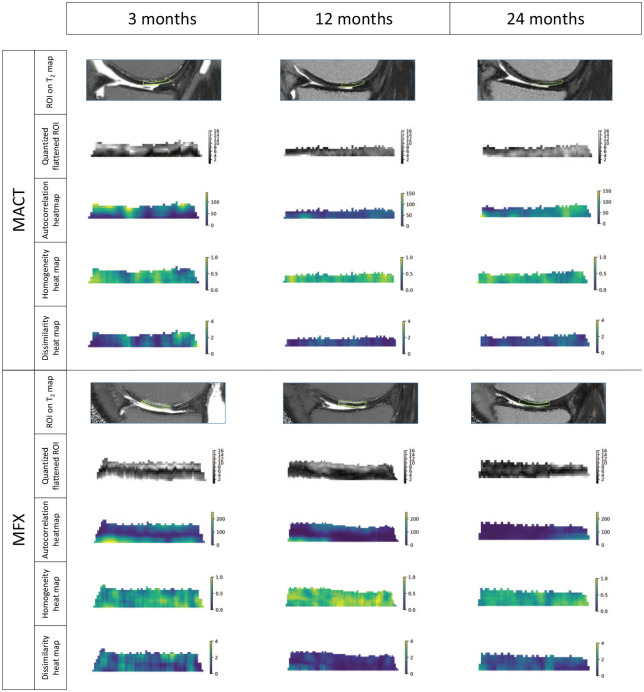
Visualization of quantized and rotated regions of interest (ROIs) and calculated features heatmaps. Value of each voxel is calculated based on surrounding pixels selected by a 5 × 5-pixel sliding window with a step of length 1. Features values for each voxel were calculated in eight directions (0°, 45°, 90°, 135°, 180°, 225°, 270°, and 315°) and averaged. These visualizations represent local features variation, and the visual change might not correspond to the overall ROI values analyzed.

However, we did identify intra-group changes over the various time-points, as visible in **Fig. 3**. Bulk T_2_ decreased significantly only in the repair tissue of the MACT group (3M vs 12M, 3M vs 24M) and the MFX group (3M vs 12M, 3M vs 24M, 12M vs 24M). Repair tissue texture developed significantly over time almost exclusively in the MACT group, MFX repair tissue texture remained the same during the 24-month period, except for information measure between 3 and 24 months (*P* = 0.016). This is visible in **Fig. 3**, where the overall texture of MFX did not change, although the overall T_2_ values decreased, particularly the hydration of the superficial layer. The change of MACT texture is clearly visible. The Wilcoxon signed-rank test revealed significant differences in autocorrelation, correlation, homogeneity, information measure, information measure of correlation 2, sum average, sum of squares, and sum variance. These features are measures of gray-level homogeneity or correlation of gray levels. The features that are linked to the presence of edges or disorderliness (e.g., contrast or dissimilarity) were not significantly different between time-points. Nevertheless, our focus on analyzing dissimilarity stems from its correlation with the MOCART score and its correlation with other features related to disorderliness. Models indicated a reduction in gray-level homogeneity and a decline in correlations between gray levels in the repair tissue over time, for example a decrease in autocorrelation (3M-12M: Δ = 25.1, *P* = 0.002; 3M-24M: Δ = 23.25, *P* = 0.004) and homogeneity (3M-24M: Δ = 0.04, *P* = 0.004). However, disorderliness increased, particularly dissimilarity (3M/24M: ratio = 0.83, *P* = 0.01). For detailed formulas and an explanation of features, please refer to the Appendix. Initially, when the repair tissue was highly hydrated, differences between the highest and lowest T_2_ voxel values were large, with similar T_2_ values falling into one quantization bin, which resulted in larger areas of uniform gray levels. When water content decreased and a finer texture with a smaller variance of T_2_ values was revealed, quantization resulted in greater local disparity in gray levels (**Fig. 4**). Therefore, homogeneity decreased and dissimilarity increased. Heatmaps (**Fig. 4**) depict local feature variations calculated with different parameters than the overall ROI feature values. Nonetheless, they illustrate how T_2_ variations translate into shades of gray and subsequent feature values.

Similar trends in texture feature changes between 12 and 24 months were estimated in a prior study that examined the MACT treatment of larger lesions (5.5 ± 1.9 cm^2^) using different MACT product. More specifically, autocorrelation (-5.14, CI [-10.84, 0.56], *P* = 0.077), homogeneity (-0.02, CI [-0.04, -0.01], *P* = 0.001), and cube of correlation (-0.06, CI [-0.09, -0.03], *P* < 0.001).^36^ In reference to the three-month follow-up of this cohort, we estimated the difference in autocorrelation (-25.10, CI [-39.35, -10.85], *P* = 0.001) at 12M and (-23.25, CI [-37.50, -9.00], *P* = 0.002) at 24M. For homogeneity, differences were estimated to be (-0.07, CI [-0.11, -0.03], *P* < 0.001) at 12M and (-0.12, CI [-0.16, -0.08], *P* < 0.001) at 24M. Last, for the cube of correlation, the estimation was (-0.01, CI [-0.09, 0.06], *P* = 0.70) at 12M and (-0.10, CI [-0.18, -0.03], *P* = 0.007) at 24M. This clearly shows the robustness of the method across different cohorts and different MACT products.

In our analysis, we found that with regard to T_2_, repair tissue differs from adjacent and reference cartilage only at 3 months in both types of procedures. However, in the case of MACT, texturally, the ROI types differed significantly over the course of the study, but we would like to draw attention to the nonsignificant differences between repair and adjacent tissue. At 3M, autocorrelation (*P* = 0.875) and sum average (*P* = 0.732), and at 24M (*P* = 0.280), homogeneity (*P* = 0.101), correlation (*P* = 0.183), and information measure (*P* = 0.790) show no significant differences either. This demonstrates that texturally adjacent tissue is close to repair tissue, confirming the results of the aforementioned study of different MACT product.^36^ It is worth noting that, at 24M, information measure was significantly different between adjacent and reference tissue (*P* = 0.002), suggesting adjacent tissue can be between repair and reference tissue texture-wise. When looking at MFX, there were significant differences between repair and reference tissue, particularly at the 12-month mark, and for autocorrelation and sum average at all three time-points. However, there were almost no significant differences between repair and adjacent tissue, as well as adjacent and reference tissue pairings. Exceptions include repair and adjacent tissue at 3 months in terms of correlation and information measure (*P* = 0.001 and *P* = 0.023, respectively). This shows that MACT affects the whole cartilage and the maturation process is probably not finished at 24M when compared with MFX.

The effect of reference tissue in the linear mixed-effects models has a similar direction and roughly equal magnitude when compared with a previous study. Namely, autocorrelation (ours: 29.6, *P* = 0.001; previous:^36^ 49.11, *P* < 0.001), homogeneity (ours: -0.12, *P* < 0.001; previous:^36^ −0.07, *P* = 0.002), and cube of correlation (ours: -0.41, *P* < 0.001; previous:^36^ −0.17, *P* = 0.015), once again pointing to the method’s robustness.

Gradual changes in tissue texture can be captured noninvasively and conveniently, thanks to the availability of T_2_ mapping protocols on clinical scanners and GLCM libraries. However, the maturation of MACT grafts into hyaline-like tissue is a gradual process, spanning up to five years.^42^ In our study, there is apparent development in MACT structure after two years not only in terms of the reduction of increased water content and swelling compared with MFX, but also in terms of T_2_ redistribution (change in texture), implying tissue reorganization. However, we were unable to distinguish whether these changes in features reflect hyaline-like cartilage or fibrocartilage because no histological assessment of biopsies was performed, as this would have put a significant burden on the patient. Although a link between texture and histology was previously shown in the case of OA,^38^ more studies of cartilage repair will be needed to validate correlations between GLCM features and the histological characteristics of repair tissue.

Our study has several limitations. The number of cases is relatively low (27 MACT and 10 MFX patients). Therefore, some differences, although important, might not be significant in this study, but the direction of the change is comparable to other cartilage repair trials. Texture analysis was conducted on three consecutive slices rather than utilizing a 3D approach due to the nonzero slice distance in multi-echo spin-echo T_2_ mapping. However, utilization of the same calculation pipeline ensured comparability between studies. Last, the absence of histological evaluation prevented us from establishing a direct connection between textural features and cartilage maturation, highlighting the need for such analysis in future studies.

## Conclusion

In conclusion, GLCM analysis of cartilage repair tissue has proven to be robust across multiple trials and MACT products. Even though there was no significant difference in parameters between MACT and MFX, significant tissue development over time was uncovered by the GLCM analysis in the case of MACT only, allowing noninvasive monitoring of graft maturation. Last, GLCM features have the potential to become the parameter that links microscopic structure with morphological appearance.

## Supplemental Material

sj-docx-1-car-10.1177_19476035241313047 – Supplemental material for Texture Analysis of Cartilage Repair Tissue Maturation: Comparison of Two Cartilage Repair Methods and Correlation with MOCART 2.0 Supplemental material, sj-docx-1-car-10.1177_19476035241313047 for Texture Analysis of Cartilage Repair Tissue Maturation: Comparison of Two Cartilage Repair Methods and Correlation with MOCART 2.0 by Veronika Janacova, Pavol Szomolanyi, Diana Sitarcikova, Alexandra Kirner, Siegfried Trattnig and Vladimir Juras in CARTILAGE
