# Supplementary material for: Texture Analysis of Cartilage Repair Tissue Maturation: Comparison of Two Cartilage Repair Methods and Correlation with MOCART 2.0
Source: Cartilage. 2025 Jan 29;17(2):164–82. doi: 10.1177/19476035241313047 (PMC11780626; doi:10.1177/19476035241313047)
Supplement: sj-docx-1-car-10.1177_19476035241313047 – Supplemental material for Texture Analysis of Cartilage Repair Tissue Maturation: Comparison of Two Cartilage Repair Methods and Correlation with MOCART 2.0 [file sj-docx-1-car-10.1177_19476035241313047.docx]

**Appendix A.** Analyzed GLCM features

| **Feature** | **Formula** | **Description** | **Ref.** |
| --- | --- | --- | --- |
| *Autocorrelation* | $\sum_{i=1}^{N} \sum_{j=1}^{N} (i\cdot j)p(i,j)$ | Represents the extent of pattern repetition and periodically changes, depending on the repetitiveness of the pattern. | [1] |
| *Correlation* | $\sum_{i=1}^{N} \sum_{j=1}^{N} \frac{(i\cdot j)p(i,j)-\mu_{x}\mu_{y}}{\sigma_{x}\sigma_{y}}$ | Correlation is calculated differently from the other texture measures. As a result, it gives different information, and therefore, is more independent of other features. Its values are always between -1 and +1 and it provides information about the predictable and linear relationship between the two neighboring pixels. | [2] |
| *Homogeneity* | $\sum_{i=1}^{N} \sum_{j=1}^{N} \frac{p(i,j)}{1+{(i-j)}^{2}}$ | It is a measure of the homogeneity of an image, with more uniform gray levels, resulting overall in a higher overall value. | [2] |
| *Dissimilarity* | $\sum_{i=1}^{N} \sum_{j=1}^{N} \left\vert i-j \right\vert p(i,j)$ | Dissimilarity is a measure of the local intensity variation. A larger value correlates with a greater disparity in intensity values among neighboring pixels. | [2] |
| *Difference entropy* | $-\sum_{k=0}^{N-1} p_{x-y}(k) log p_{x-y}(k)$ | Difference entropy belongs to measures related to orderliness and is similar to entropy. It is a measure of the randomness/variability in value differences between neighboring pixels. | [2] |
| *Sum average* | $\sum_{k=2}^{2N} p_{x+y}\left( k \right)k$ | Average sum of gray levels. Measures the relationship between occurrences of pairs with lower intensity values and occurrences of pairs with higher intensity values. | [2] |
| *Information measure (of correlation)* | $\frac{HXY - HXY1}{max\left\{ HX,HY \right\}}$ | Information measure assesses the correlation between the probability distributions of *i* and *j* (quantifying the complexity of the texture), using mutual information. In the case where the distributions are independent, there is no mutual information and the result will therefore be 0. In the case of complete dependence it will result in −1. | [2] |

$p\left( i,j \right),$ the normalized co-occurence matrix is equal to $\frac{P(i,j)}{\sum P(i,j)}$ with $P(i,j)$ being the $(i,j)$-the entry of the computed GLCM;

N is the total number of gray levels in the image

$\mu_{x}$, $\mu_{y}$ and $\sigma_{x}$, $\sigma_{y}$ denote the mean and standard deviations of the row and column sums of the GLCM, respectively

The gray level difference distribution is defined as $p_{x-y}\left( k \right)= \sum_{i=1}^{N} \sum_{j=1}^{N} p\left( i,j \right)$, $k = 0,1,2...,N-1$ and $\left| i-j \right| = k$

The gray level sum distribution is defined as$p_{x+y}\left( k \right)= \sum_{i=1}^{N} \sum_{j=1}^{N} p\left( i,j \right)$, $k = 2,3...,2N$ and $i+j = k$

$HXY = -\sum_{i=1}^{N} \sum_{j=1}^{N} p\left( i,j \right)\log_{2}\left( p\left( i,j \right)+\varepsilon\right)$ is the joint entropy of the normalized co-occurence matrix$p\left( i,j \right)$

$HXY = -\sum_{i=1}^{N} \sum_{j=1}^{N} p\left( i,j \right)\log_{2}\left( p_{x}(i)p_{y}(j)+\varepsilon\right)$ is the conditional entropy

$HX = -\sum_{i=1}^{N} \sum_{j=1}^{N} p_{x}\left( i \right)\log_{2}\left( p_{x}\left( i \right)+\varepsilon\right)$ is the entropy of $p_{x}\left( i \right)$

$HY = -\sum_{i=1}^{N} \sum_{j=1}^{N} p_{y}\left( j \right)\log_{2}\left( p_{y}\left( j \right)+\varepsilon\right)$ is the entropy of $p_{y}\left( j \right)$

$p_{x}\left( i \right) =\sum_{j=1}^{N} p\left( i,j \right)$ represents the marginal row probabilities

$p_{y}\left( j \right) =\sum_{i=1}^{N} p\left( i,j \right)$ represents represents the marginal column probabilities

[1] L.-K. Soh and C. Tsatsoulis, ‘Texture analysis of SAR sea ice imagery using gray level co-occurrence matrices’, *IEEE Transactions on Geoscience and Remote Sensing*, vol. 37, no. 2, pp. 780–795, Mar. 1999, doi: 10.1109/36.752194.

[2] R. M. Haralick, K. Shanmugam, and I. Dinstein, ‘Textural Features for Image Classification’, *IEEE Transactions on Systems, Man, and Cybernetics*, vol. SMC-3, no. 6, pp. 610–621, 1973, doi: 10.1109/TSMC.1973.4309314.

**Appendix B.** Descriptive statistics of parameters
